# Shotgun proteomic analysis of *Yersinia ruckeri* strains under normal and iron-limited conditions

**DOI:** 10.1186/s13567-016-0384-3

**Published:** 2016-10-06

**Authors:** Gokhlesh Kumar, Karin Hummel, Maike Ahrens, Simon Menanteau-Ledouble, Timothy J. Welch, Martin Eisenacher, Ebrahim Razzazi-Fazeli, Mansour El-Matbouli

**Affiliations:** 1 Clinical Division of Fish Medicine, Department for Farm Animals and Veterinary Public Health, University of Veterinary Medicine, Vienna, Austria; 2 VetCore Facility for Research/Proteomics Unit, University of Veterinary Medicine, Vienna, Austria; 3 Medizinisches Proteom-Center, Ruhr-University Bochum, Bochum, Germany; 4 National Center for Cool and Cold Water Aquaculture, Kearneysville, USA

## Abstract

*Yersinia ruckeri* is the causative agent of enteric redmouth disease of fish that causes significant economic losses, particularly in salmonids. Bacterial pathogens differentially express proteins in the host during the infection process, and under certain environmental conditions. Iron is an essential nutrient for many cellular processes and is involved in host sensing and virulence regulation in many bacteria. Little is known about proteomics expression of *Y. ruckeri* in response to iron-limited conditions. Here, we present whole cell protein identification and quantification for two motile and two non-motile strains of *Y. ruckeri* cultured in vitro under iron-sufficient and iron-limited conditions, using a shotgun proteomic approach. Label-free, gel-free quantification was performed using a nanoLC-ESI and high resolution mass spectrometry. SWATH technology was used to distinguish between different strains and their responses to iron limitation. Sixty-one differentially expressed proteins were identified in four *Y. ruckeri* strains. These proteins were involved in processes including iron ion capture and transport, and enzymatic metabolism. The proteins were confirmed to be differentially expressed at the transcriptional level using quantitative real time PCR. Our study provides the first detailed proteome analysis of *Y. ruckeri* strains, which contributes to our understanding of virulence mechanisms of *Y. ruckeri*, and informs development of novel control methods for enteric redmouth disease.

**Electronic supplementary material:**

The online version of this article (doi:10.1186/s13567-016-0384-3) contains supplementary material, which is available to authorized users.

## Introduction

Enteric redmouth disease (ERM) is one of the most important diseases of salmonids, and causes significant economic losses in commercial aquaculture worldwide [1]. The disease is caused by *Yersinia ruckeri*, a Gram-negative rod-shaped enterobacterium. This bacterium has been reported in Europe, North and South America, Middle East and China [1–4]. *Yersinia ruckeri* strains are divided into two biotypes: biotype 1 strains are motile and lipase secretion positive, whereas strains of biotype 2 are non-motile and test negative for lipase [5]. In the past, the majority of epizootic outbreaks in salmonids were caused by biotype 1 strains, against which an effective vaccine was developed [6]. However, biotype 2 strains have recently been responsible for outbreaks in both native and vaccinated rainbow trout (*Oncorhynchus mykiss*) in Europe and North America, suggesting that this biotype was able to overcome the protection granted by motile strains vaccines [3, 7, 8].

Iron is an essential nutrient for microorganisms and occurs as ferrous and ferric oxidation states. It influences cell composition, secondary metabolism, enzyme activity, host cell interactions and pathogenicity of bacteria [9]. During reduced availability of iron pathogens express differentially regulated proteins, several of which are involved in invasion and pathogenicity activities. Iron homeostasis systems and virulence mechanisms have not been thoroughly investigated in *Y. ruckeri* and little is known about its protein expression. The expression of outer membrane proteins (OMPs) of *Y. ruckeri* isolates was examined by Davies [10] using SDS-PAGE, who observed four OMP bands at 72, 69.5, 68 and 66 kDa under iron-limited conditions. Tinsley et al. [11] also used SDS-PAGE to show induction in OMPs of *Y. ruckeri* isolates at 90 and 100 kDa, under iron-restricted conditions; these proteins were not identified by mass spectrometry. An alternative protein analysis technology has emerged: label-free, gel-free shotgun proteomics, and this has been used in *Y. pestis* to identify whole cell proteins [12, 13]. Recently, a reference proteome map of *Y. pestis* has been created, and is leading to an understanding of the pathogenesis of this bacterium [12]. Additionally, differentially expressed proteins of *Y. pestis* and *Y. enterocolitica* have been identified and quantified under iron-limited conditions using both 2D-PAGE and MALDI TOF/TOF MS [14, 15].

The aim of the present study was to identify and quantify the whole cell proteomic expression profiles of biotype 1 and biotype 2 strains of *Y. ruckeri* grown in vitro, under normal and iron-limited conditions using a label-free, gel-free shotgun proteomics approach. This study represents one of the first descriptive and comparative proteomic approaches of motile and non-motile *Y. ruckeri* strains in response to iron-limited conditions.

## Materials and methods

### *Yersinia ruckeri* strains

**Figure 1:**
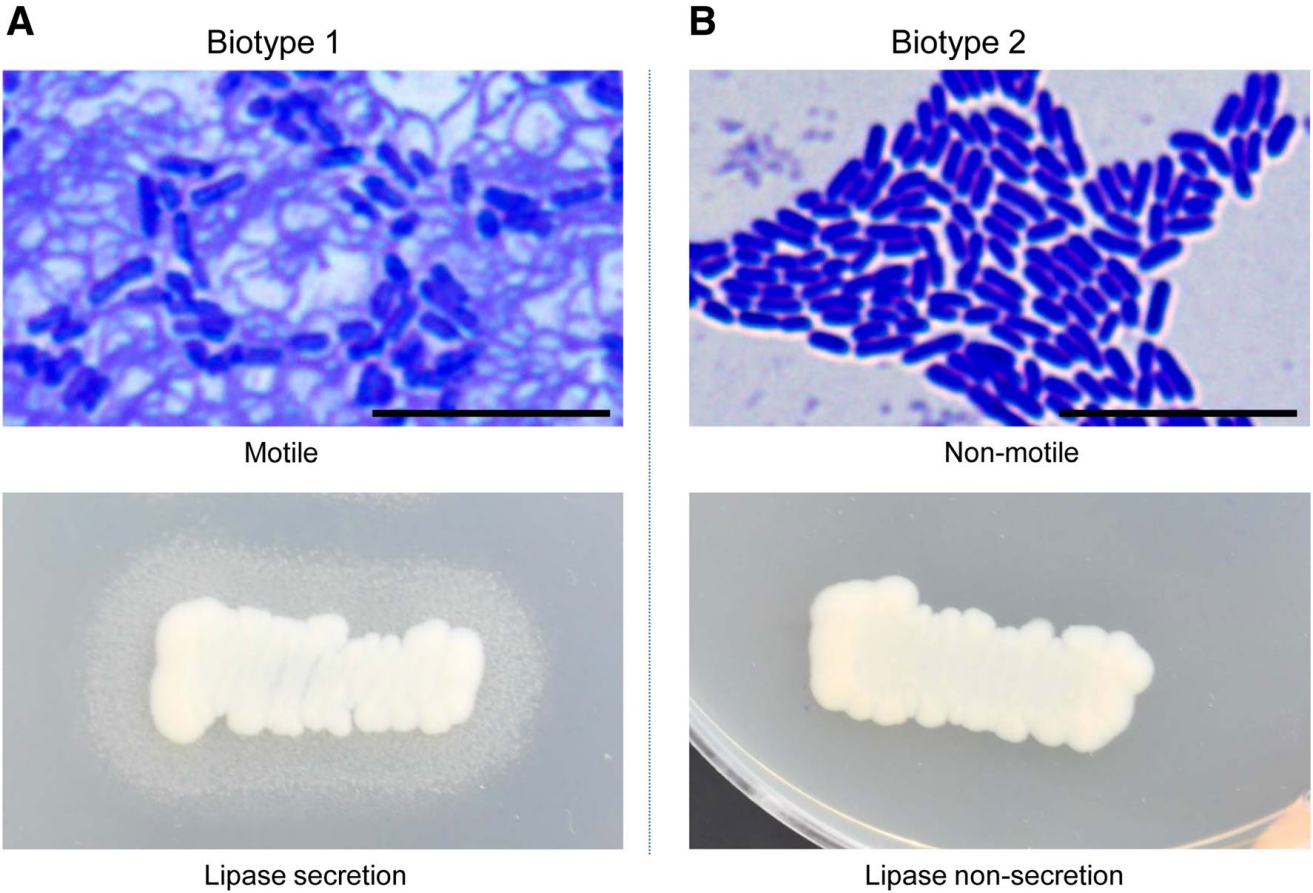
**Flagellar motility and lipase secretion of** ***Y. ruckeri*** **biotype 1 and 2 strains.** Motility and lipase examinations were performed at 24 and 48 h, respectively. **A** Biotype 1 strain shows flagellar motility and lipase secretion, **B** Biotype 2 strain does not show flagella and lipase secretion. Flagella were examined by staining using a Flagella Stain (BD) and phospholipase activity was determined on tween 80-tryptic soy agar plates. Scale bar 10 µm.

Four strains (SP-05, CSF007-82, 7959-11 and YRNC-10) of *Y. ruckeri* were used for deep proteomic analysis. Strains SP-05 and 7959-11 were obtained from our bacterial repository at the University of Veterinary Medicine, Vienna, Austria. The other two strains, CSF007-82 and YRNC-10, were obtained from the National Center for Cool and Cold Water Aquaculture, Kearneysville, West Virginia, USA. These strains were tested for serotype, flagella motility and phospholipase activity [3] and their identity confirmed by PCR [16]. Strains, SP-05 and CSF007-82, belong to serotype 1 and biotype 1 (motile and lipase positive, Figure 1A), while strains 7959-11 and YRNC-10 belong to serotype 1 and biotype 2 (non-motile and lipase negative, Figure 1B).

### Culture conditions

Bacterial strains were grown on tryptic soy agar for 48 h at 22 °C. A single colony of each strain was inoculated into 10 mL of tryptic soy broth [Casein peptone (17 g), dipotassium hydrogen phosphate (2.5 g), glucose (2.5 g), sodium chloride (5 g), soya peptone (3 g) per liter, Sigma, Germany] and incubated for 17 h at 22 °C with shaking (150 rpm). These starter cultures were then diluted with fresh sterile tryptic soy broth to an optical density (OD 600) of 0.10 ± 0.05. Five hundred microlitres of the diluted starter cultures were inoculated in duplicates, into 25 mL of tryptic soy broth with or without iron chelator, 2,2′-bipyridyl, MW 156.18 g/mol (Sigma, Germany). Iron depletion was attained by the addition of 100 μM of 2,2′-bipyridyl to the broth. Cultures were grown overnight at 22 °C with shaking (150 rpm) until the late log phase. Cells were harvested by centrifugation at 4000 rpm for 10 min at 4 °C, then washed three times with phosphate buffered saline containing bacterial protease inhibitor cocktail (Sigma, Germany) and stored at −80 °C.

### Protein extraction and digestion

Bacterial pellets were resuspended in 800 µL pre-cooled denaturing lysis buffer (7 M urea, 2 M thiourea, 4% CHAPS and 1% DTT) containing bacterial protease inhibitor cocktail. Bacterial cell suspensions were disrupted by sonication on ice for 20 cycles of 10 s pulse-on and 30 s pulse-off. The lysates were centrifuged at 14 000 rpm for 30 min at 4 °C and supernatants were collected. Total protein concentration of each lysate was determined colorimetrically with a NanoDrop 2000c (Thermo Fisher Scientific, USA) spectrophotometer using a Pierce 660 nm Protein Assay according to the manufacturer’s instructions.

Protein digestion was performed using the standard two-step in-solution digestion protocol for Trypsin/LysC-Mix according to the user manual (Promega, USA). In summary, 10 µg of proteins was diluted with 8 M urea in 50 mM Tris–HCl (pH 8.0) to a total volume of 10 µL. Proteins were reduced in 5 mM dithiothreitol (final concentration) for 30 min at 37 °C and alkylated with 15 mM iodoacetamide for 30 min at 25 °C. Trypsin/LysC mix was added in a ratio of 25:1 protein to protease (w/w). The first digestion step was performed for 4 h at 37 °C under high urea concentration. To enable protease activity of trypsin, urea was diluted afterwards with 50 mM Tris–HCl (pH 8.0) to a concentration below 1 mol/L. The second digestion step was performed for 8 h at 37 °C. Finally, digested samples were acidified with concentrated trifluoroacetic acid.

### NanoLC-MS/MS analysis

Tryptic peptides were separated by a nano liquid chromatography system (Dionex Ultimate 3000 RSLC, Thermo Fisher Scientific, USA) and analyzed with a high-resolution hybrid triple quadrupole time of flight mass spectrometer (TripleTOF 5600+, Sciex, USA) coupled via a nano-ESI interface.

Samples were pre-concentrated and desalted with a 5 mm Acclaim PepMap µ-Precolumn (300 µm inner diameter, 5 µm particle size, and 100 Å pore size) (Dionex, Thermo Fisher Scientific, USA). Samples were loaded and desalted using 2% acetonitrile (ACN, ChemLab, Belgium) in LC–MS grade water (Optima, Thermo Fisher Scientific, USA). 0.05% TFA was used as a mobile phase with a flow rate of 5 µL/min. Three microliter injection volumes (corresponding to 370 ng digested protein absolute on column) were used per injection.

Peptide separation was performed on a 25 cm Acclaim PepMap C18 column (75 µm inner diameter, 2 µm particle size, and 100 Å pore size) with a flow rate of 300 nL/min. The gradient started with 4% B (80% ACN with 0.1% formic acid) and increased to 35% B in 120 min. It was followed by a washing step with 90% B. Mobile phase A consisted of LC-MS grade water with 0.1% formic acid.

For information dependent data acquisition (IDA runs), MS1 survey scans were collected in the range of 400–1500 m/z. The 25 most intense precursors with charge state 2–4 that exceeded 100 counts per second, were selected for fragmentation for 250 ms. MS2 product ion scans were collected in the range of 100–1800 m/z for 110 ms. Precursor ions were dynamically excluded from reselection for 12 s.

For quantitative measurements, data independent SWATH (Sequential Window Acquisition of all Theoretical spectra) technology based on MS2 quantification was used [17, 18]. Peptides were fragmented in 35 fixed fragmentation windows of 20 Da in the range of 400–1100 Da with an accumulation time of 50 ms in TOF MS mode and 80 ms in product ion mode. The nano-HPLC system was operated by Chromeleon 6.8 (Dionex, USA) and the MS by Analyst Software 1.6 (Sciex, USA).

### Data processing, quantification and statistical evaluation

IDA raw data was processed with ProteinPilot Software version 5.0 (Sciex, USA) for re-calibration and database searches. The UniProt database (Release 04_2015) was restricted to *Yersinia ruckeri*, but contained possible contaminants from the growth medium, e.g. *Glycine max* (Soybean) and bovine caseins. Mass tolerance in MS mode was set with 0.05 and 0.1 Da in MSMS mode for the rapid recalibration search, and 0.0011 Da in MS and 0.01 Da in MSMS mode for the final search. The following sample parameters were applied: trypsin digestion, cysteine alkylation set to iodoacetamide, search effort set to rapid ID. False discovery rate analysis (FDR) was performed using the integrated tools in ProteinPilot. Global false discovery rate was set to <1% on protein level.

IDA identification results were used to create the SWATH ion library with the MS/MS (ALL) with SWATH Acquisition MicroApp 2.0 in PeakView 2.2 (both Sciex, USA). Peptides were chosen based on a FDR rate <1%, excluding shared and modified peptides. Up to 6 peptides per protein and up to 6 transitions per peptide were used.

MarkerView 1.2.1 (Sciex, USA) was used for calculation of peak areas of SWATH samples after retention time alignment and normalization using total area sums. Resulting protein lists were then used for visualization of data after principal component analysis (PCA) in form of loadings plots and score plots to get a first impression of the overall data structure, and to assess variability between technical and biological replicates.

To determine differentially regulated proteins, statistical evaluation was performed in R programming language [19]. Raw peak areas after normalization to total area sums were log_2_-transformed to approach a normal distribution. On a logarithmic scale, technical replicates were aggregated by arithmetic mean before application of statistical tests. This procedure is equivalent to the application of a hierarchical model in the subsequent ANOVA, as the same number of technical replicates was measured per biological replicate.

For each growing condition, differential expression of proteins in each *Y. ruckeri* strain was assessed using one-way ANOVA for each protein. To adjust for multiple testing, the method of Benjamini and Hochberg [20] was used to control the FDR. Differences were considered significant if adjusted *p*-values were smaller than the significance level of *α* = 0.001. For those proteins, Tukey’s honest significant difference (HSD) method was applied as post hoc test to assess the significance of the pairwise comparisons. Protein expression was considered differential if the adjusted *p* value was below *α* and the absolute fold change was at least three (fold change < −3 or > +3).

In a second approach, significant differences between the two growing conditions were examined separately for each strain with unpaired two-sample *t* tests. All resulting *p* values were adjusted for multiple comparisons by FDR [20]. Candidates of interest were chosen based on a *p* value of lower than 0.05 (corresponding to −log_10_ (*p*) of 1.3) and a fold change < −3 or > +3 (corresponding to log_2_ (rm) of <− 1.6 or >1.6). Results were summarized as Volcano plots (ratio of means vs. adjusted *p* value). Fold changes of all significant candidates were listed as tables for each comparison. Computations related to the statistical test procedures were performed in R. Afterwards, venn diagrams were used as a tool for data visualization.

### GO annotation of differentially expressed proteins

We used software tool for rapid annotation of proteins (STRAP version 1.5) for classification of biological process, cellular component and molecular function of differentially expressed proteins of *Y. ruckeri* [21].

### qRT-PCR analysis

Total RNA was extracted from *Y. ruckeri* strains grown under normal and iron-limited conditions using an RNeasy Mini Kit (Qiagen) and included an on-column DNase digestion step. cDNA was synthesized using an iScript cDNA Synthesis Kit (BIO-RAD) with 1 µg total RNA according to the user’s manual.

PCR primers specific for the six selected genes were designed using NCBI Primer-BLAST software (Additional file 1) and optimized using gradient PCRs to determine the annealing temperature and primer concentration. qPCRs were performed in a final volume of 20 µL, which contained 4 µL of 1:20 fold diluted cDNA, 0.4 µM of each primer, 1X SYBR Green Supermix and sterile distilled water. After 5 min of cDNA denaturation at 95 °C, 38 cycles were performed at 95 °C for 30 s, 57 °C for 30 s and 72 °C for 30 s. Each qPCR was performed in biological replicates with three technical reactions using CFX96 Touch Real-Time PCR detection system (BIO-RAD).

Recombination protein A, signal recognition particle protein and 16S rRNA genes were tested to determine the suitable stability of them as reference genes. All raw Cq values from qPCR were used to assess the output of the four computational programs (geNorm, Normfinder, BestKeeper, and delta Ct method) using web-based RefFinder software tools [22]. Based on RefFinder, recombination protein A and signal recognition particle protein were found to be most stable reference genes across normal and iron-limited samples, and thus were used for normalization of target genes. Relative quantity values of each sample of each target gene were normalized with values of two reference genes (recombination protein A and signal recognition particle protein) using CFX Manager Software version 3.1 (BIO-RAD) in normalized expression mode (∆∆Cq). Then, normalized values of each sample were used for statistical analysis and differences between both growing conditions were analyzed using *t*-tests for samples with Bonferroni α-correction. All statistical analyses were conducted with SPSS version-20 software. The expression levels were considered statistically significant if *p* values were <0.05.

## Results

### Bacterial growth under iron-limited condition

Growth curves of *Y. ruckeri* strains were determined in tryptic soy broth. Three strains (CSF007-82, 7959-11 and YRNC-10) were shown to have growth culture patterns similar to each other. However, the yield of strain SP-05 was slightly lower compared to the other three strains. The iron chelator, 2,2′-bipyridyl reduced the growth and yield of all strains as the final optical density reading of iron-limited strains was always lower than under iron-sufficient conditions (Additional file 2).

### Label-free quantification of proteins by SWATH technology

Identified proteins at FDR 1% with more than one peptide of each strain under normal and iron-limited conditions are given in Additional file 3. Results of *t* test comparisons (*p* < 0.05) between normal and iron-limited conditions for each strain are provided in Additional file 4. Proteins determined to be expressed differently among the four *Y. ruckeri* strains according to ANOVA and Tukey HSD analysis (*p* < 0.001) with a fold change of < −3 or > +3 are listed in supplementary data (Additional files 5 and 6).

Additional file 7 shows a PCA graphical overview of the complex proteomics data of strains. PCA score plots confirmed high reproducibility of biological replicates by grouping the replicates together. The score plots of all strains under both growth conditions suggested that strain SP-05 in both conditions differs from the three strains (CSF007-82, 7959-11 and YRNC-10). However, these three strains showed minor proteomic differences between each other. Principal component scores for PC1 and PC2 were 63.5 and 14.7% (Additional file 7A) under normal condition and 60.4 and 9.8% (Additional file 7B) under iron-limited condition, respectively, showed that a major part of the overall variation in the data was captured in the first two principal components. Volcano plots (Additional file 8) were used for joint visualization of effect size and corresponding significance of the differentially expressed proteins.

### Differentially expressed proteins of *Y. ruckeri* strains under iron-limited condition

In *Y. ruckeri*, we identified 1395 proteins under normal and 1447 proteins under iron-limited growth conditions (Additional file 3). Sophisticated statistical evaluation revealed a total number of 61 differentially expressed proteins within the four analyzed *Y. ruckeri* strains (Tables 1, 2, 3). Of these, 35 proteins were upregulated and 26 proteins were downregulated. We found one uncharacterized (K7M2F1_SOYBN) soybean protein as a contaminant in our protein samples because of tryptic soy broth medium, which contains soya peptone.

**Table 1:** **Differentially expressed proteins of** ***Y. ruckeri*** **strains (SP-05, CSF007-82, 7959-11 and YRNC-10)**

UniProt accession number	Ambiguous accession number	Protein	Function	SP-05	CSF007-82	7959-11	YRNC-10
Iron ion binding							
C4UM10_YERRU	A0A0A8VIR2_YERRU	Iron(III)-binding periplasmic protein	Iron ion binding	22.3*	26*	25.5*	28.5*
C4UKR8_YERRU	A0A085U4N5_YERRU	Iron ABC transporter substrate-binding protein	Iron binding protein	17.4	35.0*	25.6*	18.8*
C4UFX7_YERRU	A0A085U819_YERRU	Periplasmic chelated iron-binding protein yfeA	Iron ion binding	4.1*	3.8*	3.3*	3.1
C4UFT8_YERRU	A0A085U7X7_YERRU	Hemin receptor	Iron receptor activity	4.4*	1.2	1.1	−1.0
C4UHA0_YERRU	A0A085U6V7_YERRU	Bacterioferritin	Ferric iron binding	−3.0*	−10.2*	−9.5*	−12.5*
C4UGG9_YERRU	A0A085UAC6_YERRU	Iron-sulfur cluster assembly scaffold protein IscU	Iron-sulfur cluster binding	−3.7*	−3.8*	−3.6*	−3.6*
Transport							
C4UFT9_YERRU	A0A085U7X8_YERRU	Hemin transport protein hemS	Iron ion transport	96.2*	20.6*	17.3	13.6*
C4UH16_YERRU	A0A085U729_YERRU	Ferrous iron transport protein A	Ferrous iron import	4.2	6.8*	7.9*	6.6*
C4UH15_YERRU	A0A0A5FIY5_YERRU	Ferrous iron transport protein B	Ferrous iron transport	−1.1	2.5	5.1*	2.1
C4UFU0_YERRU	A0A085U7X9_YERRU	Hemin-binding periplasmic protein hmuT	Iron transport	8.6	2.5	2.5	3.4*
A0A094SXA9_YERRU	A0A085UBN5_YERRU	Fe(3+) ions import ATP-binding protein FbpC	Ferric-transporting ATPase activity	2.7	3.4	3.1*	2.5
C4UKR5_YERRU	A0A0A8VKF9_YERRU	TonB-dependent receptor plug	Transport/Receptor activity	7.7	9.5*	4.5*	1.3
C4UJT7_YERRU	A0A085U605_YERRU	Bacterial extracellular solute-binding s, 3 family protein	Transporter activity	2.5	2.4	3.2*	3.1*
A0A094TKG1_YERRU	A0A085U820_YERRU	ABC transporter family protein	Transmembrane transport	3.9*	3.0	2.9	2.5
A0A094SUN9_YERRU	A0A085U4N3_YERRU	ABC transporter family protein	Transmembrane transport	5.4	4.7*	5.8*	6.8*
A0A094SST2_YERRU	A0A085U3C5_YERRU	ABC transporter family protein	Transmembrane transport	3.2*	2.8	4.8	2.9
C4UK69_YERRU	A0A085U6M5_YERRU	Sec-independent protein translocase protein TatA	Transporter activity	−1.4	−1.6	−6.2	−7.0*
C4UG32_YERRU	A0A085U4W0_YERRU	NADH dehydrogenase (Quinone), D subunit	Transport	−3.5*	−3.0*	−1.6	−1.7
C4UHI5_YERRU	A0A085U5C9_YERRU	Acriflavine resistance protein A	Drug transmembrane transport	−3.7*	−1.9	−1.3	−2.6
C4UMG6_YERRU	A0A085U472_YERRU	Cytochrome d ubiquinol oxidase subunit 1	Electron transport coupled proton transport	−3.7*	−1.6	1.2	−1.4
C4UEW4_YERRU	A0A085U499_YERRU	d-Galactose-binding periplasmic protein	Carbohydrate transport	−1.2	−3.3*	−2.6	−2.2

Differentially expressed proteins of *Y. ruckeri* strains under iron-limited conditions (Normal versus iron-limited conditions) were sorted by function. * denotes statistically significant difference according to both *t*-test with FDR-adjusted *p* value <0.05 and fold change < −3 or > +3.

**Table 2:** **Differentially expressed proteins of** ***Y. ruckeri*** **strains (SP-05, CSF007-82, 7959-11 and YRNC-10)**

UniProt accession number	Ambiguous accession number	Protein	Function	SP-05	CSF007-82	7959-11	YRNC-10
Siderophore							
C4UM82_YERRU	A0A085U3B8_YERRU	Enterobactin synthetase component B	Siderophore biosynthesis	23.4*	18.7*	19.2*	10.5*
C4UM84_YERRU	A0A0A8VG89_YERRU	Enterobactin synthetase component C	Biosynthetic process	4.4*	5.2*	6.2*	4.8
A0A0A8VLD7_YERRU	A0A085U3B7_YERRU	2,3-dihydro-2,3-dihydroxybenzoate dehydrogenase of siderophore biosynthesis	Siderophore biosynthesis	13.8*	19.2*	16.5*	22.7*
C4UM94_YERRU	A0A085U3D0_YERRU	Ferrichrysobactin receptor	Siderophore transport	11.2*	10.1*	14.4*	14.9*
A0A0A8VMF7_YERRU	A0A085U3B9_YERRU	2,3-dihydroxybenzoate-AMP ligase	Siderophore biosynthesis	2.8	3.7	5.2*	3.9*
Copper							
C4UHL3_YERRU	A0A0A8VB61_YERRU	Copper-exporting P-type ATPase A	Copper-exporting ATPase activity	3.8*	3.5*	2.8	2.3
C4UI46_YERRU	A0A085U353_YERRU	Blue copper oxidase CueO	Copper ion binding	2.2	3*	2.7	2.8
Oxidation reduction							
C4UGW6_YERRU	A0A0A8VE22_YERRU	Superoxide dismutase Mn	Superoxide dismutase activity	4.6	5.6*	5.5*	3.5*
C4UFR4_YERRU	A0A085U7V3_YERRU	Superoxide dismutase Fe	Superoxide dismutase activity	−28.6*	−13.9*	−14.3*	−12.3*
C4ULL1_YERRU	A0A085U5T3_YERRU	Glutaredoxin	Electron carrier activity	16.6	3.5*	3.8*	7.4*
C4UJD2_YERRU	A0A085U8M0_YERRU	NAD(P)H nitroreductase ydjA	Oxidoreductase	2.5	3.2*	2.8	2.7
C4UFI5_YERRU	A0A085U7N0_YERRU	NAD(P) transhydrogenase subunit beta	NADP binding	2.1	3.4*	2.2	1.1
C4UKC5_YERRU	A0A085U882_YERRU	FAD-binding 9 siderophore-interacting domain protein	Oxidoreductase	4.2	8.6	5.7*	5.8*
C4UNK9_YERRU	A0A085U9C9_YERRU	Peptide methionine sulfoxide reductase msrA	Peptide-methionine (S)-S-oxide reductase activity	−1.1	*3.3* *	2.0	2.0
C4UMV1_YERRU	A0A085U9H1_YERRU	Fumarate reductase flavoprotein subunit	FAD binding	−3.3*	−2.5	−2.2	−2.0
C4UKS8_YERRU	A0A085U4P6_YERRU	Molybdopterin guanine dinucleotide-containing S/N-oxide reductases family protein	Molybdenum ion binding	−4.3*	−2.6	−2.4	−1.9
Flagella							
A0A094V4E9_YERRU	A0A085U8W7_YERRU	Flagellin	Structural molecule activity	1.1	−4.2*	1.4	1.5
A0A0A8VDU4_YERRU	R4NIZ0_YERRU	Flagellar biosynthesis protein FliC	Structural molecule activity	−1.1	−5.3*	1.3	−1.1
C4UKI3_YERRU	A0A085U8Z4_YERRU	Flagellar L-ring family protein	Motor activity	−1.0	−2.7	−3.8	−4.7*
A0A094TLS7_YERRU	A0A085U8Y5_YERRU	Flagellar motor switch protein FliN	Motor activity	1.1	−1.2	−3.6*	1.2

Differentially expressed proteins details as in Table 1.

**Table 3:** **Differentially expressed proteins of** ***Y. ruckeri*** **strains (SP-05, CSF007-82, 7959-11 and YRNC-10)**

UniProt accession number	Ambiguous accession number	Protein	Function	SP-05	CSF007-82	7959-11	YRNC-10
Metabolic							
C4UF95_YERRU	A0A085U4I3_YERRU	2,3-bisphosphoglycerate-dependent phosphoglycerate mutase	Glycolytic process	3.8*	3.6*	3.2*	3.1*
C4UMV0_YERRU	A0A085U9H2_YERRU	Succinate dehydrogenase iron-sulfur subunit	Glycolytic process/iron-sulfur cluster binding	−3.3*	−1.9	−1.9	−1.9
C4UH25_YERRU	A0A0A8VEE9_YERRU	Phosphoenolpyruvate carboxykinase	Gluconeogenesis	−3.2*	1.3	−4.0	−3.0
C4UIX8_YERRU	A0A085U3P6_YERRU	2,3-bisphosphoglycerate-independent phosphoglycerate mutase	Glycolytic process	−4.0*	−3.5*	−3.7*	−3.6*
C4UN43_YERRU	A0A085U407_YERRU	Fumarate hydratase class I, aerobic	Fumarate hydratase activity	−4.6*	−2.5	−2.4	−2.7
A0A0A5FJV0_YERRU	A0A085U4U1_YERRU	Catalase	Peroxidase activity	−6.2*	−2.7	−2.4	−2.7
Amino acid biosynthesis							
A0A0A8VHV7_YERRU	A0A085U7L2_YERRU	Aromatic-L-amino-acid decarboxylase	Amino acid metabolic process	1.3	1.9	4.4*	3.4*
C4UIX0_YERRU	A0A085U3N7_YERRU	Periplasmic protein CpxP	Protein misfolding	4.4*	1.8	2.6	1.9
C4UM91_YERRU	A0A0A8VLE4_YERRU	Amino acid adenylation domain protein	Amino acid activation for nonribosomal peptide biosynthetic process	1.1	2.0	3.9*	2.4
C4UL33_YERRU	A0A085UBI2_YERRU	FKBP-type 16 kDa peptidyl-prolyl *cis*–*trans* isomerase	Protein folding	−1.7	−1.0	3.3	5.0*
A0A094STG7_YERRU	A0A085U807_YERRU	50S ribosomal protein L35	rRNA binding	−2.3	−3.4	−1.9	−3.6*
C4UFW5_YERRU	A0A085U806_YERRU	50S ribosomal protein L20	rRNA binding	−4.1*	−1.9	1.0	−1.6
Others							
A0A094SMR6_YERRU	A0A085U3C8_YERRU	MbtH-like family protein	Nonribosomal peptide formation	14.6	5.6*	12.5	7.7
C4UJX4_YERRU	A0A085U549_YERRU	Uncharacterized protein	Putative exported protein	4.9*	1.4	1.3	1.5
A0A094SSK4_YERRU	A0A085UBK3_YERRU	Probable phosphoglycerate mutase GpmB	Phosphoglycerate mutase activity	−3.1*	−1.1	−2.0	−1.4
C4UF96_YERRU	A0A085U4I2_YERRU	PsiF repeat protein	None predicted	−1.9	−5.2*	−4.5	−1.1
C4UEV3_YERRU	A0A085U4A9_YERRU	Molybdopterin biosynthesis protein moeB	Molybdopterin cofactor biosynthetic process	1.1	−1.2	−2.3	−4.1*
C4UP53_YERRU	A0A085U2Q2_YERRU	Phosphopantetheine attachment site family protein	Phosphopantetheine binding	−1.8	−1.2	−1.7	−3.2*
C4UJI8_YERRU	A0A0A8VHP1_YERRU	Peptidase S49 family protein	Serine-type endopeptidase activity	−1.0	−2.3	−2.9	−4.1*
C4UFK9_YERRU	A0A085U7Q2_YERRU	Uncharacterized protein	Putative lipoprotein	−1.2	1.1	−1.0	4.8*

Differentially expressed proteins details as in Table 1.

As can be seen in Tables 1, 2, 3, most of the upregulated proteins were related to iron ion binding and transport and in particular to siderophore, oxidoreductase and glycolysis activities. The rest of the proteins were related to copper, nonribosomal peptide formation, protein folding and lipoprotein. Similarly, most of the downregulated proteins were related to transport, flagellar motility, oxidoreductase, iron binding, translation and glycolysis activities.

**Figure 2:**
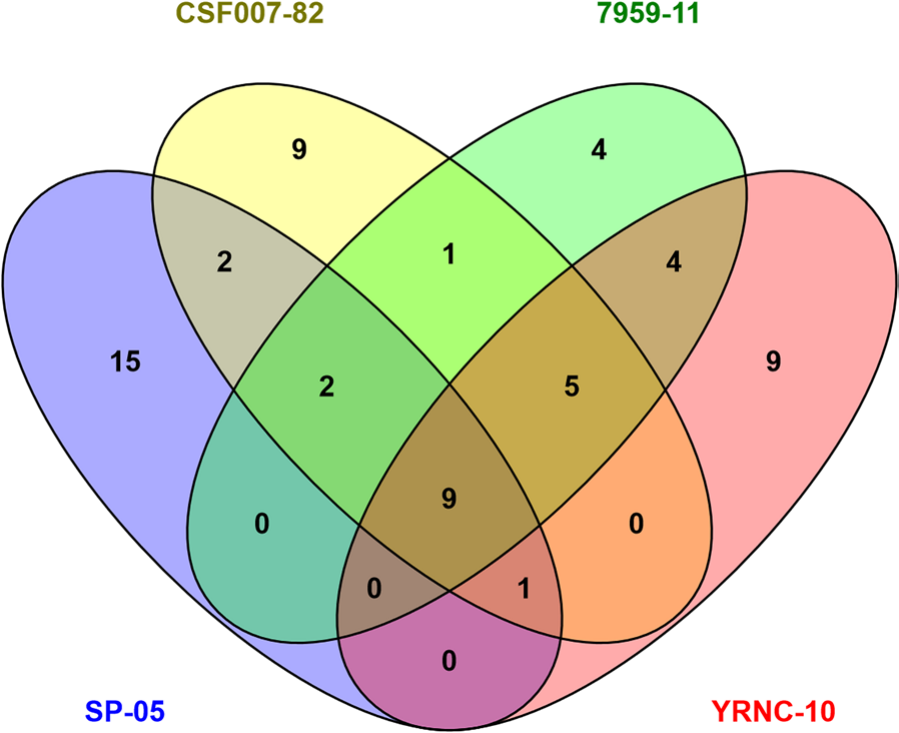
**Venn diagram showing the number of differentially expressed proteins identified in** ***Y. ruckeri*** **strains**. The number of unique or shared proteins of each strain is indicated in each set or subset.

Fifteen proteins in SP-05, nine proteins in CSF007-82, four proteins in 7959-11 and nine proteins in YRNC-10 were uniquely differentially expressed (Figure 2). When comparing all strains, we noted some proteins that were upregulated by fold changes between 10 and 96: iron(III)-binding periplasmic protein (YfuA), iron ABC transporter substrate-binding protein (YiuA), periplasmic chelated iron-binding protein (YfeA), hemin transport protein (HemS), ferrous iron transport protein A (FeoA), ferrichrysobactin receptor, enterobactin synthetase component B & C (EntB and EntC) and 2,3-dihydro-2,3-dihydroxybenzoate dehydrogenase of siderophore biosynthesis (EntA) proteins, which were highly expressed in both motile and non-motile *Y. ruckeri* strains.

### GO annotation of differentially expressed proteins

**Figure 3:**
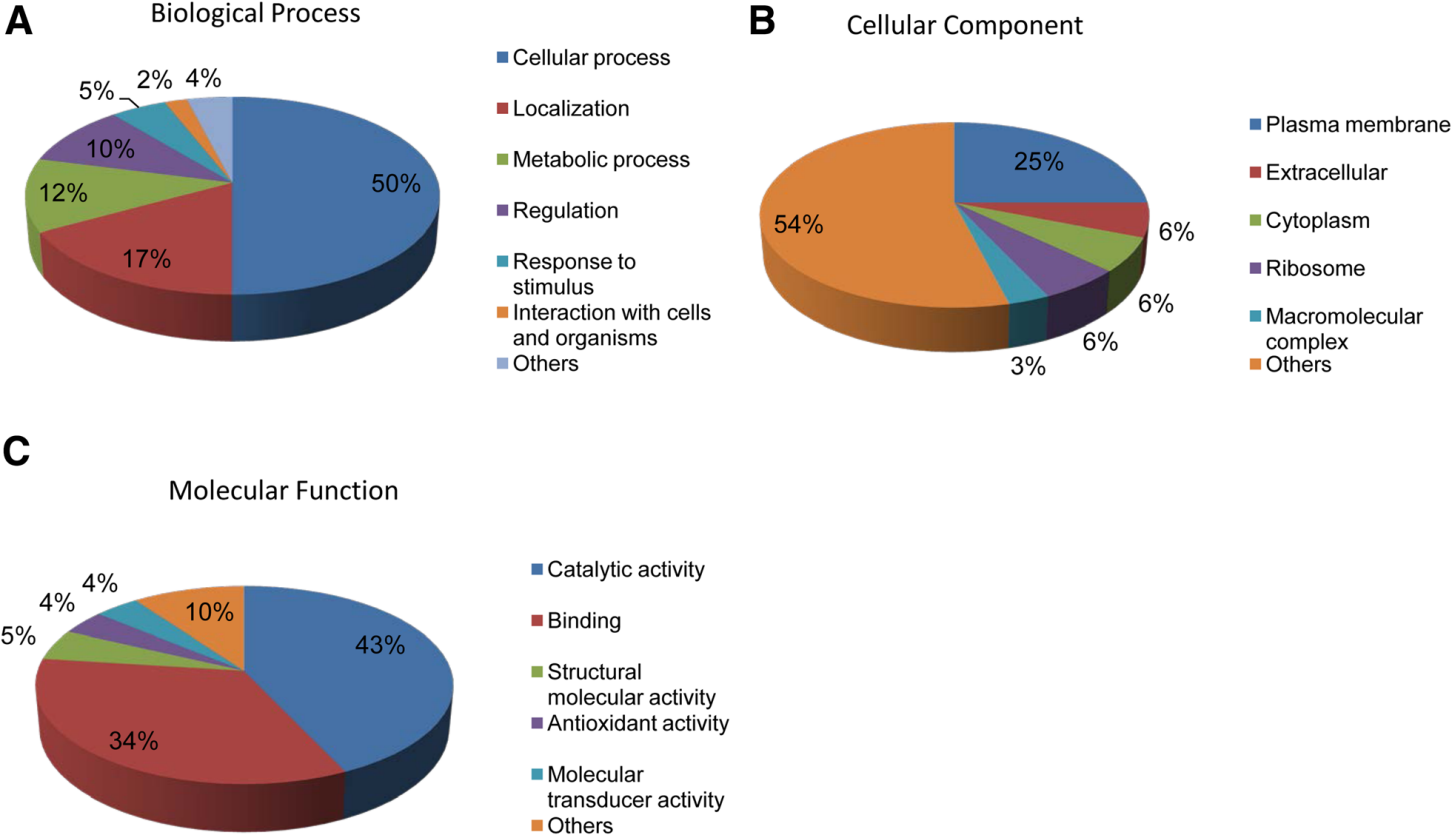
**Classification of differentially expressed proteins of** ***Y. ruckeri***. Proteins were classified by GO terms for biological processes, cellular components and molecular functions using STRAP. **A** Biological process, **B** cellular component, and **C** molecular function.

Differentially expressed proteins of *Y. ruckeri* were classified by Gene Ontology terms for biological processes, cellular components and molecular functions using the STRAP software. The identified proteins were associated with cellular process (50%), localization (17%), metabolic process (12%), regulation (10%), response to stimulus (5%), and interaction with cells and organisms (2%) (Figure 3A). Most of the differentially expressed proteins were localized in the plasma membrane (25%), extracellular (6%) and cytoplasm (6%) (Figure 3B). Differentially expressed proteins were involved in catalytic (43%), binding (34%), structural molecule (5%) and molecular transducer activities; functions of 10% of the proteins remained others (Figure 3C).

### qRT-PCR

**Figure 4:**
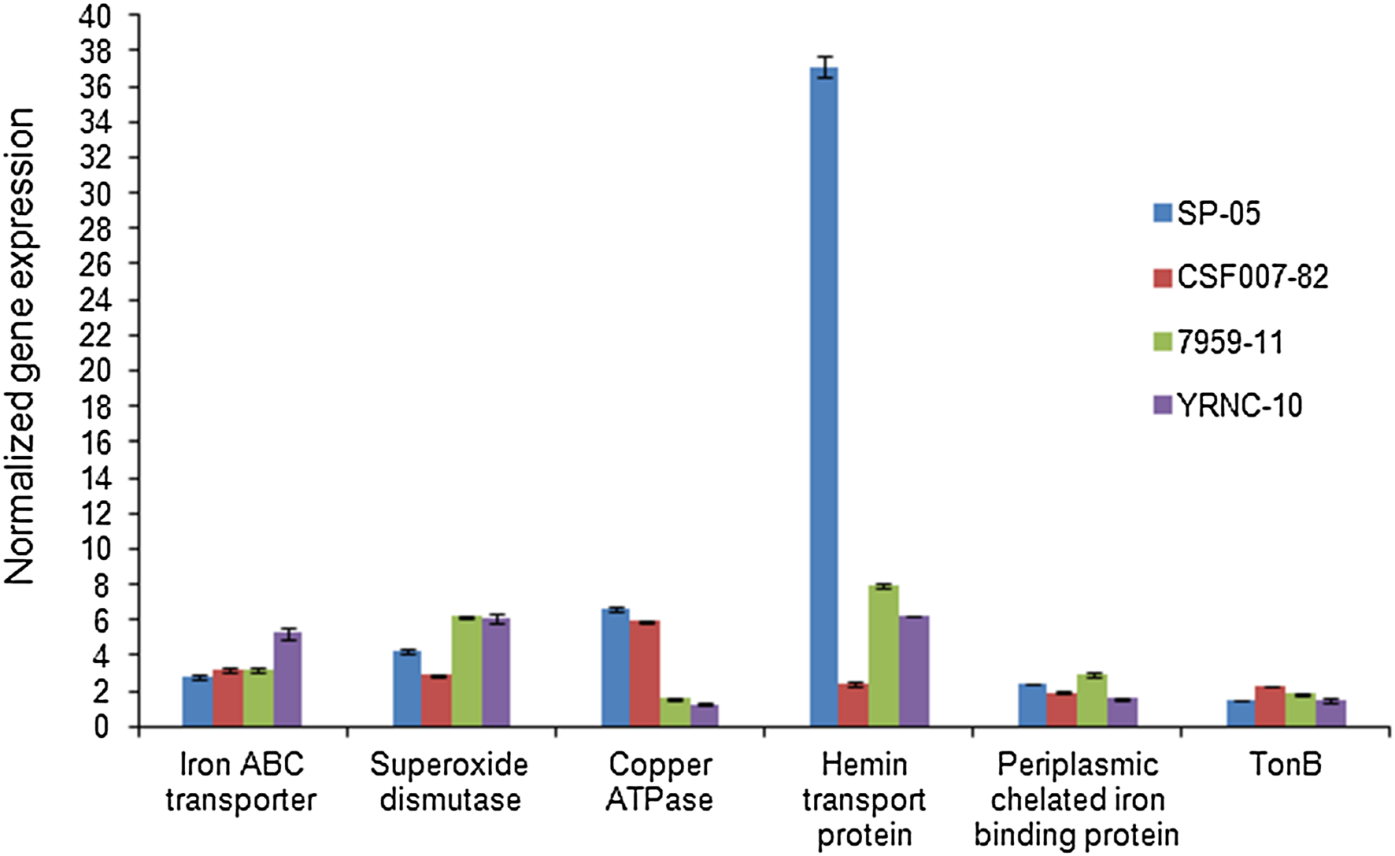
**Relative expression profiles of** ***Y. ruckeri*** **strains.** Quantitative real-time PCR showing relative expression profiles of different genes coding for differentially expressed proteins under iron-limited culture conditions. Relative gene expression changes of each gene were determined in biological replicates by calculating the mean normalized expression values of each *Y. ruckeri* strains. Error bars indicate standard deviation.

Differentially expressed proteins identified by shotgun proteomics analysis were validated at the transcriptional level by qRT-PCR (Figure 4). We selected six genes coding for differentially expressed proteins. The candidate proteins were selected on the basis of their differential expression as representative of iron binding protein, superoxide metabolic process, copper-exporting P-type ATPase, iron ion transport and siderophore biosynthesis. Selected genes were overexpressed (*p* < 0.0001) in the iron-limited samples, relative to the control samples (normal growth condition).

## Discussion

Label-free shotgun proteomics is a straightforward, fast, cost effective and broadly applicable approach to quantify proteins [23]. This approach has been used successfully for the identification and quantification of proteins in *Y. pestis*, the causative agents of bubonic plague [12, 13]. In the present study, we identified and quantified whole cell proteins of biotype 1 and biotype 2 *Y. ruckeri* strains using a label-free, gel-free shotgun proteomic method. We identified 1395 proteins in *Y. ruckeri*, with strains having between 1077 and 1161 proteins under normal growth conditions. This number of proteins is consistent with that reported in other members of the genus: 1074 proteins were identified in *Y. pestis* KIM5 isolated from human [12]; 1421 in *Y. pestis* 91 001 isolated from mice [13]; 1074–1078 in *Y. pestis* Yp1945/Yp2126 isolated from fleas [24]. In the present study, 61 differentially expressed proteins (35 up- and 26 down-regulated) were identified in vitro in *Y. ruckeri* strains (FDR-adjusted *p* value <0.05 and fold change < −3 or > +3) in response to iron-restriction conditions. The function of these proteins included iron binding, molecular transduction, as well as catalytic and structural molecule activities.

In both motile and non-motile *Y. ruckeri* strains, we observed strong upregulation of iron-binding proteins (YfuA, YiuA and YfeA; 3.3- to 35-fold) and iron-binding transporters (HemS and Feo: 6.6- to 96.2-fold) (Tables 1, 2, 3). Previous work has shown in *Y. pestis* in a mouse model that Yfu and Yiu function as part of an iron uptake system, but are not involved in virulence [25]. The YfeA system, which transports iron and manganese, is required for the full virulent phenotype of *Y. pestis* [26]. Upregulation (1.6- to 4.5-fold) of iron-binding proteins YfuA, YiuA and YfeA, has been reported in *Y. pestis* and *Y. enterocolitica* in response to iron starvations [14, 15]. This is consistent with the results from our in vitro *Y. ruckeri.* Hemin transport protein HemS is involved in the uptake of hemin-containing iron. Hemolysin activator protein was identified as an in vivo induced extracellular cytolysin in *Y. ruckeri* [27] and was implicated in the virulence and cytotoxicity of *Y. ruckeri* [28]. A ferrous iron transport system plays a role in the acquisition of ferrous iron during intracellular growth of the bacterium and is composed of three proteins, FeoA, FeoB and FeoC [29]. Both FeoA and FeoB are essential components of the iron acquisition system in *Y. pestis* [30]. Upregulation of FeoA and FeoB proteins under iron-starvation conditions suggests that the Feo system is involved in the iron acquisition system of *Y. ruckeri* during the infection process, however, its significance for pathogenesis is unknown. Based on the results of the present study, it appears that the iron binding proteins/transporters are upregulated in response to iron-scarcity. Because iron is often the limiting factor during the infectious process, this has likely clinical implications regarding the survival of the bacterium inside the host and the establishment of disease by both motile and non-motile *Y. ruckeri* strains.

We found that ferrichrysobactin and TonB-dependent iron-binding receptors were upregulated 4.5- and 14.9-fold, respectively, in *Y. ruckeri* strains under iron-limited conditions. Notably, in *Y. ruckeri*, both of these receptors have been shown to be upregulated during the infection process in fish [27]. TonB-dependent receptors are outer membrane receptors of Gram-negative bacteria involved in active transport of a number of molecules, including the uptake of siderophores [9, 31]. The role of the *tonB* gene has been examined in *Y. pestis*, where utilization of siderophore and uptake of hemin are TonB-dependent [32]. Siderophores are small, high-affinity iron chelating molecules secreted by bacteria, and are transported into the periplasm of cells by TonB-dependent receptors [9, 31]. We found for the first time, that the siderophore biosynthesis proteins EntA and EntB are upregulated in *Yersinia* species in response to iron-limited conditions. Upregulation (17.95-fold) of TonB has been observed in *Edwardsiella ictaluri* during in vitro iron restriction [33] and in *Aeromonas salmonicida* during in vivo infection [34]. Similarly, strong upregulation (4.4- or 23.4-fold) of siderophore biosynthesis proteins (EntA, EntB and EntC) were observed in iron-starved *Y. ruckeri* cells. This suggests that iron is a limiting factor and that iron-binding receptors are required for the survival of *Y. ruckeri* during the infection in the fish host.

Superoxide dismutases (SODs) represent one of the major defense mechanisms of cells against oxidative stress, by catalyzing conversion of superoxide radicals [35]. SODs are classified based on the bound metal ion co-factor, for example: iron (Fe-SOD), manganese (Mn-SOD), copper/zinc (Cu–Zn-SOD) and nickel (Ni-SOD) [36, 37]. We found 3.5- to 5.6-fold upregulation of Mn-SOD (SodA), and 12.3- to 28.6-fold downregulation of Fe-SOD (SodB) in *Y. ruckeri* strains under iron-limited conditions. The regulation of superoxide dismutases has been linked to iron metabolism in another important fish pathogen, *A. salmonicida* subsp. s*almonicida*: Ebanks et al. [38] observed expression of SodA under both iron restriction and *fur*::KO mutant growth conditions, and demonstrated involvement of the ferric uptake regulator in the pathogenicity of the bacterium in fish. The *sodA* gene has been shown to contribute to the pathogenicity of many other bacteria, including *Y. enterocolitica* and *Streptococcus agalactiae*, during host invasion, using a mouse model [35, 37].

We observed downregulation of catalase in iron-depleted *Y. ruckeri* cells, but the magnitude of the change was not significant (< −threefold). Catalase contains four porphyrin heme groups and catalyzes hydrogen peroxide into water and oxygen, thereby protecting the bacterium from this reactive oxygen species [39]. Downregulation of catalase may be due the depletion of porphyrin heme groups during iron-limited conditions.

We observed upregulation of copper-exporting P-type ATPase (>3.5-fold) in motile *Y. ruckeri* strains SP-05 and CSF007-82, but saw no significant expression in non-motile strains under iron-limited conditions. This expression was validated by qRT-PCR to confirm the results of shotgun proteomic analysis. Copper-exporting P-type ATPase A is involved in copper export from the cytoplasm to the periplasm, and it is known that copper metabolism in bacteria is linked to that of iron, and that some siderophores interact with copper [40]. A link between copper and siderophores has been shown in *Escherichia coli* [41]. We are the first to report the upregulation of copper-exporting P-type ATPase in *Yersinia* species in response to iron-limited conditions, however the relevance of this to pathogenicity is unknown as there is a paucity of knowledge on copper metabolism in *Yersinia* species.

Under iron-limited conditions, we observed 3.1- to 5.8-fold upregulation of enzymes involved in glycolysis (2,3-bisphosphoglycerate-dependent phosphoglycerate mutase) and oxidoreduction (glutaredoxin and FAD-binding 9 siderophore-interacting domain protein), suggesting an increased role of these enzymes in metabolic processes when the cells are starved of iron. Furthermore, we saw down-regulation other metabolic enzymes (fumarate reductase, fumarate hydratase, phosphoenolpyruvate carboxykinase and molybdopterin). This was expected, as these enzymes are dependent on iron-sulfur clusters or other iron cofactors, and is consistent with previous observations of downregulation of metabolic proteins in iron-starved *Y. pestis* cells [14].

Cellular iron depletion can suppress expression levels of iron-dependent proteins [9, 31]. We observed strong downregulation of iron-dependent proteins, including bacterioferritin (−3 to −12.5-fold) and iron-sulfur cluster assembly scaffold protein IscU (−3.6 to −3.8-fold) in *Y. ruckeri* strains under iron starvation conditions. Bacterioferritin is a main cytoplasmic iron storage protein and IscU provides iron for iron-sulfur cluster assembly [9, 31]. These observations are consistent with a shift in bacterial metabolic activities to iron-independent biochemical pathways when the supply of iron ion is limited.

We observed −3.6 to −5.3-fold downregulation of flagellar proteins including FliC, FlgH and FliN in *Y. ruckeri* strains under iron-limited conditions. Previous mutational research conducted by Evenhuis et al. [42] showed that the absence of flagellar motility does not affect *Y. ruckeri* virulence, and that flagellin expression is not required for production of innate immune response in fish [43], as suggested by the clinical importance of non-motile isolates of the bacterium [44].

Shotgun proteomics revealed minor protein differences among motile and non-motile *Y. ruckeri* strains under normal and iron-limited growth conditions, consistent with observations by Huang et al. [45, 46] who used pulsed-field gel electrophoresis and fatty acid profiles. In the present study, PCA score plots (Additional file 7) highlight major protein differences between the European motile strain (SP-05) and USA motile strain (CSF007-82). These differences may be due to high genetic diversity and epidemic population structure as seen by multilocus sequence typing [47].

In conclusion, this is the first detailed description of differentially expressed proteins in *Y. ruckeri* strains cultured in iron limited conditions, as revealed by shotgun proteomic analysis. It is likely that the changes in expression of iron-binding proteins, iron ion transporters/receptors, siderophore biosynthesis proteins and manganese-binding superoxide dismutase play an important role in the survival of *Y. ruckeri* inside the host. These findings might provide the basis for the development of novel control methods for enteric redmouth disease in fish.

## Additional files

10.1186/s13567-016-0384-3 **List of quantitative real-time PCR primers.** PCR primers specific for the selected genes were designed using NCBI Primer-BLAST software and used in this study.

10.1186/s13567-016-0384-3 **Growth curves of** ***Yersinia ruckeri*** **strains.** Duplicate bacterial strains were grown in tryptic soy broth under normal and iron-limited conditions at 22 °C. Growth was monitored at different time points by determining the optical density at 600 nm. Error bars indicate standard deviation. ILC: Iron limited culture.

10.1186/s13567-016-0384-3 **Total number of identified proteins of** ***Y. ruckeri*** **strains.** Number of proteins was identified at FDR 1% with more than one peptide.

10.1186/s13567-016-0384-3 **Total number of differentially expressed proteins of** ***Y. ruckeri*** **strains (strain versus strain).** Differentially expressed proteins were assessed according to ANOVA and Tukey HSD (*p* < 0.001) with a fold change < −3 or > +3.

10.1186/s13567-016-0384-3 **Fold changes of differentially expressed proteins of** ***Y. ruckeri*** **strains compared to each other under normal culture conditions.** ANOVA was performed for UniProt database searches. * denotes statistically significant difference according to *t*-test with FDR-adjusted *p* value < 0.05 and fold change < −3 or > +3.

10.1186/s13567-016-0384-3 **Fold changes of differentially expressed proteins of** ***Y. ruckeri*** **strains compared to each other under iron-limited conditions.** ANOVA was performed for UniProt database searches. * denotes statistically significant difference according to *t* test with FDR-adjusted *p* value <0.05 and fold change < −3 or > +3.

10.1186/s13567-016-0384-3 **Principal component analysis score plots of** ***Yersinia ruckeri*** **strains.** Grouping of biological replicates shows good reproducibility of technical replicates for each sample. The score plots show that strain SP-05 differs from the three strains under both normal and iron-limited conditions, which show minor protein differences to each other. (A) PCA score plot under normal condition, (B) PCA score plot under iron-limited condition.

10.1186/s13567-016-0384-3 **Volcano plots of** ***Y. ruckeri*** **strains.** Volcano plots showing the distribution of the ratios (log_2_) versus the FDR adjusted *p* value (−log_10_) for all proteins identified under iron-limited condition. Differentially expressed proteins are shown as black circles. (A) Motile strain CSF007-82, (B) Non-motile strain 7959-11.

## Abbreviations

ERM: enteric redmouth disease
OMPs: outer membrane proteins
2D-PAGE: two-dimensional gel electrophoresis
MALDI-TOF-MS: matrix-assisted laser desorption/ionization mass spectrometry
LC-MS: liquid chromatography–mass spectrometry
FDR: false discovery rate
SWATH: sequential window acquisition of all theoretical spectra
IDA: information dependent data acquisition
PCA: principal component analysis
Feo: ferrous iron transport
SOD: superoxide dismutase
