# Supplementary material for: Shotgun proteomic analysis of Yersinia ruckeri strains under normal and iron-limited conditions
Source: Vet Res. 2016 Oct 6;47:100. doi: 10.1186/s13567-016-0384-3 (PMC5054536; doi:10.1186/s13567-016-0384-3)
Supplement: Supplementary file 1 — 10.1186/s13567-016-0384-3 List of quantitative real-time PCR primers. PCR primers specific for the selected genes were designed using NCBI Primer-BLAST software and used in this study. [file 13567_2016_384_MOESM1_ESM.doc]

**Additional file 1** **List of quantitative real-time PCR primers. PCR primers specific for the selected genes were designed using NCBI Primer-BLAST software and used in this study.**

| Primer Code | Sequence (5’-3’) | Product Size (bp) | Annealing Temp. (°C) | Accession/Gene ID number |
| --- | --- | --- | --- | --- |
| TonB F | GAAAATGGAACTCGGTGGCG | 120 | 57 | Gene ID: 25229094 |
| TonB R | AAATCCCAGCCTAACTCACCG |
| Iron ABC F | CCGTGAATGCCGCTTCAAAT | 128 | 57 | Gene ID: 25226782 |
| Iron ABC R | CCCGCCATTACGCAGTATGA |
| Superoxide dismutase F | GTTGGTACTGCAAGATGGCG | 127 | 57 | Gene ID: 25229250 |
| Superoxide dismutase R | ATGTTCCCATACGTCGAGGC |
| Copper ATPase F | AAGCGCTGGAAAATCGACCT | 142 | 57 | Gene ID: 25227830 |
| Copper ATPase R 5’ | TCCGGCTGTTGTACCCATTC |
| HemS F | AATGTTGATGCCCGAACCCT | 184 | 57 | KGA51492.1 |
| HemS R | CCCACTGGCCAAGGAATAGG |
| Periplasmic yfeA F 5’ | GCCGATCAACGCTGAAGAAC | 160 | 57 | EEQ00604.1 |
| Periplasmic yfeA R | TACACCGCCGTACTGTGAAC |
| RecA F | GACCGCTCAATGGATGTTGA | 154 | 57 | FJ717382.1 |
| RecA R | CAGAGGCGATAACCTGTAGTG |
| SRPP F | GACGGGTTTGACCTGAATGA | 150 | 57 | YRUCK0001_RS15430 |
| SRPP R | CATCCGTACCGTCACTTTGT |
