# Supplementary figures and images for: Shotgun proteomic analysis of Yersinia ruckeri strains under normal and iron-limited conditions
Source: Vet Res. 2016 Oct 6;47:100. doi: 10.1186/s13567-016-0384-3 (PMC5054536; doi:10.1186/s13567-016-0384-3)

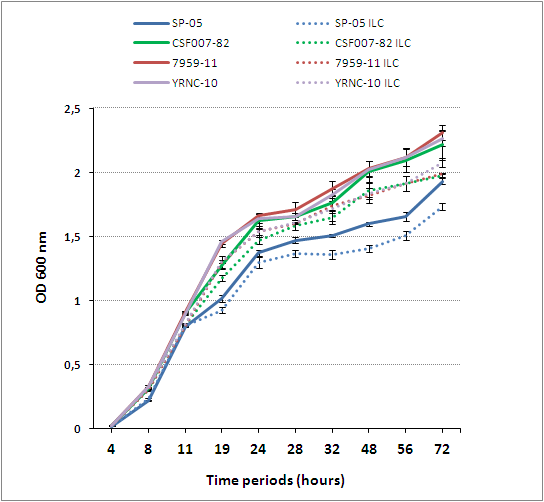

Supplement: Supplementary file 2 — 10.1186/s13567-016-0384-3 Growth curves of Yersinia ruckeri strains. Duplicate bacterial strains were grown in tryptic soy broth under normal and iron-limited conditions at 22 °C. Growth was monitored at different time points by determining the optical density at 600 nm. Error bars indicate standard deviation. ILC: Iron limited culture. [file 13567_2016_384_MOESM2_ESM.tif]

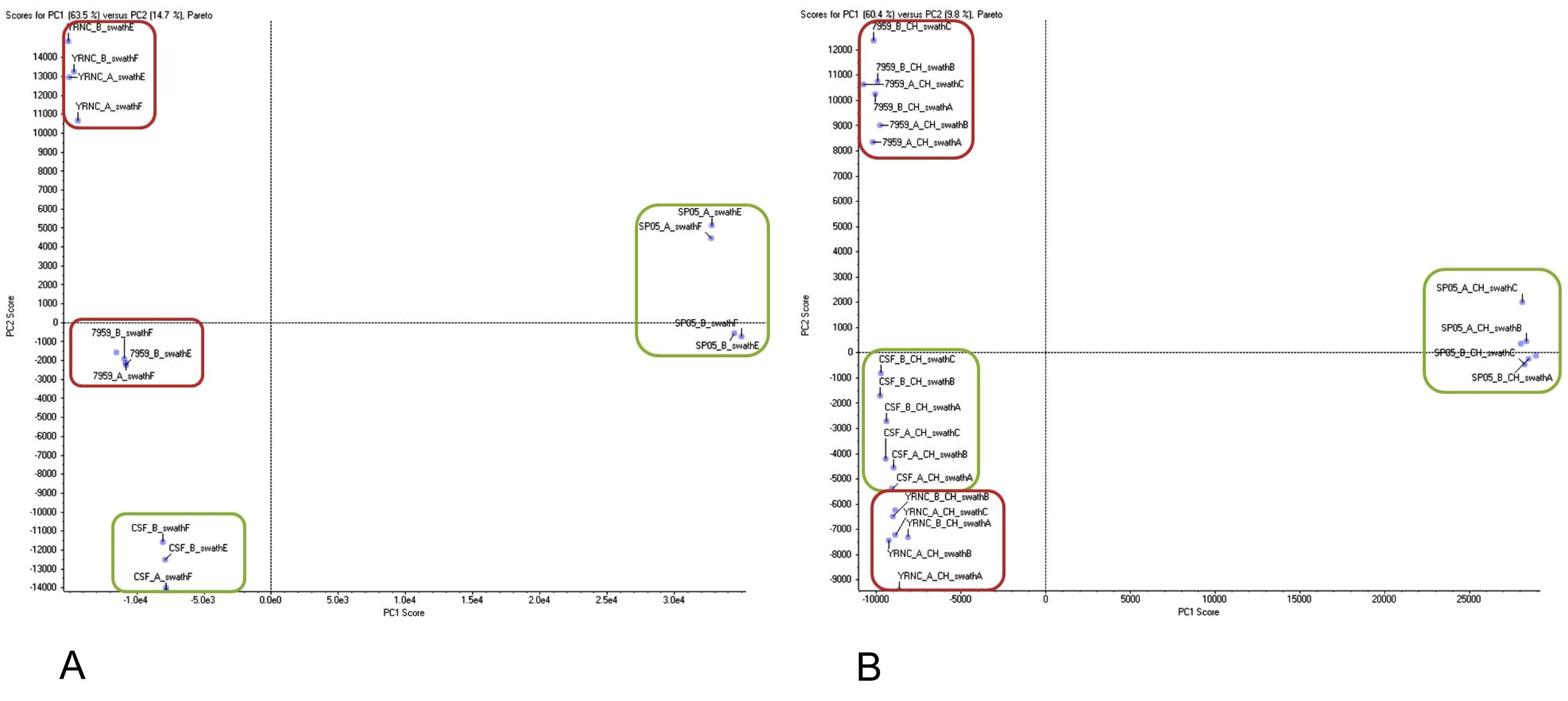

Supplement: Supplementary file 7 — 10.1186/s13567-016-0384-3 Principal component analysis score plots of Yersinia ruckeri strains. Grouping of biological replicates shows good reproducibility of technical replicates for each sample. The score plots show that strain SP-05 differs from the three strains under both normal and iron-limited conditions, which show minor protein differences to each other. (A) PCA score plot under normal condition, (B) PCA score plot under iron-limited condition. [file 13567_2016_384_MOESM7_ESM.tif]

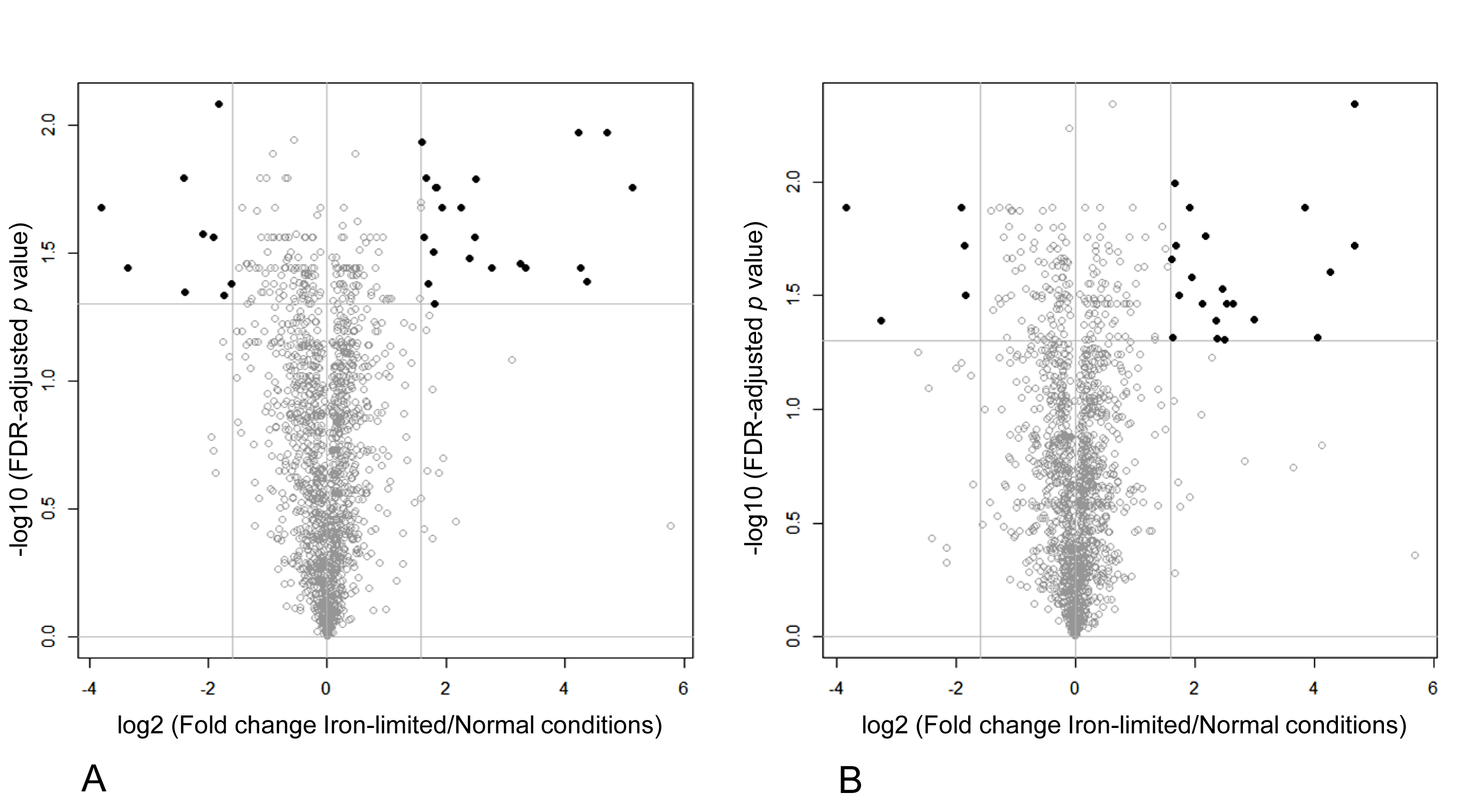

Supplement: Supplementary file 8 — 10.1186/s13567-016-0384-3 Volcano plots of Y. ruckeri strains. Volcano plots showing the distribution of the ratios (log2) versus the FDR adjusted p value (−log10) for all proteins identified under iron-limited condition. Differentially expressed proteins are shown as black circles. (A) Motile strain CSF007-82, (B) Non-motile strain 7959-11. [file 13567_2016_384_MOESM8_ESM.tif]
