# Supplementary material for: Shotgun proteomic analysis of Yersinia ruckeri strains under normal and iron-limited conditions
Source: Vet Res. 2016 Oct 6;47:100. doi: 10.1186/s13567-016-0384-3 (PMC5054536; doi:10.1186/s13567-016-0384-3)
Supplement: Supplementary file 3 — 10.1186/s13567-016-0384-3 Total number of identified proteins of Y. ruckeri strains. Number of proteins was identified at FDR 1% with more than one peptide. [file 13567_2016_384_MOESM3_ESM.doc]

**Additional file 3** **Total number of identified proteins of *Y. ruckeri* strains.** Number of proteins was identified at FDR 1% with more than one peptide.

| Biotype | Strain | Origin | Number of proteins identified under normal culture condition | Number of proteins identified under iron-limited culture condition |
| --- | --- | --- | --- | --- |
| 1 | SP-05 | Europe | 1077 | 1127 |
| 1 | CSF007-82 | USA | 1161 | 1209 |
| 2 | 7959-11 | Europe | 1134 | 1182 |
| 2 | YRNC-10 | USA | 1103 | 1167 |
