# Supplementary material for: Shotgun proteomic analysis of Yersinia ruckeri strains under normal and iron-limited conditions
Source: Vet Res. 2016 Oct 6;47:100. doi: 10.1186/s13567-016-0384-3 (PMC5054536; doi:10.1186/s13567-016-0384-3)
Supplement: Supplementary file 4 — 10.1186/s13567-016-0384-3 Total number of differentially expressed proteins of Y. ruckeri strains (strain versus strain). Differentially expressed proteins were assessed according to ANOVA and Tukey HSD (p < 0.001) with a fold change < −3 or > +3. [file 13567_2016_384_MOESM4_ESM.doc]

**Additional file 4** **Total number of differentially expressed proteins of *Y. ruckeri* strains (strain versus strain).** Differentially expressed proteins were assessed according to ANOVA and Tukey HSD (*p* < 0.001) with a fold change < −3 or > + 3.

|  | SP-05  vs.  CSF007-82 | SP-05  vs.  7959-11 | SP0-5  vs.  YRNC-10 | CSF007-82  vs.  7959-11 | CSF007-82  vs.  YRNC-10 | 7959-11  vs.  YRNC-10 |
| --- | --- | --- | --- | --- | --- | --- |
| Numbers of differentially expressed proteins (Normal condition) | 27 | 30 | 33 | 5 | 4 | 1 |
| Numbers of differentially expressed proteins (Iron-limited condition) | 36 | 39 | 39 | 4 | 2 | 2 |
