# Supplementary material for: Shotgun proteomic analysis of Yersinia ruckeri strains under normal and iron-limited conditions
Source: Vet Res. 2016 Oct 6;47:100. doi: 10.1186/s13567-016-0384-3 (PMC5054536; doi:10.1186/s13567-016-0384-3)
Supplement: Supplementary file 5 — 10.1186/s13567-016-0384-3 Fold changes of differentially expressed proteins of Y. ruckeri strains compared to each other under normal culture conditions. ANOVA was performed for UniProt database searches. * denotes statistically significant difference according to t-test with FDR-adjusted p value < 0.05 and fold change < −3 or > +3. [file 13567_2016_384_MOESM5_ESM.doc]

**Additional file 5 Fold changes of differentially expressed proteins of *Y. ruckeri* strains compared to each other under normal culture conditions. ANOVA was performed for UniProt database searches.** * denotes statistically significant difference according to *t*-test with FDR-adjusted *p*-value < 0.05 and fold change < −3 or > +3.

| **UniProt  Accession number** | **Ambiguous Accession number** | **Protein** | **SP05**  **vs.**  **CSF007** | **SP05**  **vs.**  **7959** | **SP05**  **vs.**  **YRNC** | **CSF007 vs.**  **7959** | **CSF007 vs. YRNC** | **7959 vs. YRNC** |
| --- | --- | --- | --- | --- | --- | --- | --- | --- |
| C4UK36_YERRU | A0A085U984_YERRU | Ornithine decarboxylase, inducible | **-5.5*** | **-5.5*** | **-6.1*** | -1.0 | -1.1 | -1.1 |
| C4UH25_YERRU | A0A0A8VEE9_YERRU | Phosphoenolpyruvate carboxykinase | **-123.2*** | **-4.9*** | **-4.7*** | **25.1*** | **26.2*** | 1.0 |
| C4UML3_YERRU | A0A085UAK7_YERRU | Malic enzyme, NAD binding domain protein | **-3.6*** | **-3.8*** | **-3.5*** | -1.1 | 1.0 | 1.1 |
| A0A0A8VE48_YERRU | A0A085UBQ1_YERRU | Arginine deiminase | **5.7*** | **5.2*** | **5.8*** | -1.1 | 1.0 | 1.1 |
| C4UM15_YERRU | A0A0A8VGZ8_YERRU | Asparagine synthase | **-3.9*** | 1.1 | **-4.3*** | **4.3*** | -1.1 | **-4.8*** |
| C4UFF0_YERRU | A0A0A5FMC5_YERRU | Biodegradative arginine decarboxylase | **7.9*** | **6.5*** | **6.2*** | -1.2 | -1.3 | -1.0 |
| A0A085U669_YERRU | A0A085U669_YERRU | PTS mannose transporter subunit IIAB | **-3.2*** | -2.7 | -2.9 | 1.2 | 1.1 | -1.1 |
| C4UIW0_YERRU | A0A085U3M7_YERRU | Glycerol kinase | **-4.7*** | **-6.1*** | **-5.4*** | -1.3 | -1.1 | 1.1 |
| C4UMT2_YERRU | A0A085U9J0_YERRU | Aspartate ammonia-lyase | **-4.2*** | **-4.1*** | **-5.1*** | 1.0 | -1.2 | -1.2 |
| A0A0A8VDU4_YERRU | R4NIZ0_YERRU | Flagellar biosynthesis protein FliC | -3.0 | **-109.8*** | **-78.8*** | **-36.6*** | **-26.3*** | 1.4 |
| C4ULZ9_YERRU | A0A085UBP8_YERRU | Glutamate decarboxylase | **10.4*** | **8.5*** | **6.8*** | -1.2 | -1.5 | -1.3 |
| C4ULJ6_YERRU | A0A085U5X1_YERRU | Periplasmic binding protein | -2.4 | **-3.5*** | -3.0 | -1.4 | -1.2 | 1.2 |
| C4UGR2_YERRU | A0A085U8U0_YERRU | Phosphate-binding protein PstS | 3.0 | **3.1*** | **3.4*** | 1.0 | 1.1 | 1.1 |
| C4UH73_YERRU | A0A085U745_YERRU | Glucose-1-phosphate adenylyltransferase | **5.3*** | **5.0*** | **3.8*** | -1.1 | -1.4 | -1.3 |
| C4ULZ7_YERRU | A0A085UBQ0_YERRU | Uncharacterized protein | **5.0*** | **4.9*** | **4.3*** | -1.0 | -1.2 | -1.2 |
| C4UNQ9_YERRU | A0A085UBM7_YERRU | Short chain dehydrogenase family protein | **6.2*** | **6.1*** | **6.8*** | -1.0 | 1.1 | 1.1 |
| C4UEU5_YERRU | A0A085U4B6_YERRU | DNA protection during starvation protein | **3.2*** | **3.8*** | 1.9 | 1.2 | -1.7 | -2.1 |
| C4UGB1_YERRU | A0A085U2E6_YERRU | Glucokinase | -2.4 | -2.8 | **-3.6*** | -1.2 | -1.5 | -1.3 |
| C4UG35_YERRU | A0A085U4W3_YERRU | HTH-type transcriptional regulator pecT | **-12.7*** | **-16.8*** | **-13.2*** | -1.3 | -1.0 | 1.3 |
| A0A094V4E9_YERRU | A0A085U8W7_YERRU | Flagellin | **-5.1*** | **-128.3*** | **-120.6*** | **-25.3*** | **-23.8*** | 1.1 |
| A0A094V364_YERRU | A0A085UB63_YERRU | Aminomethyltransferase | **-3.3*** | **-3.2*** | **-3.9*** | 1.0 | -1.2 | -1.2 |
| C4UJJ3_YERRU | A0A085U7G0_YERRU | Putative uncharacterized protein | **9.1*** | **7.9*** | **9.5*** | -1.2 | 1.0 | 1.2 |
| C4UHA0_YERRU | A0A085U6V7_YERRU | Bacterioferritin | **6.8*** | **5.7*** | **6.5*** | -1.2 | -1.0 | 1.1 |
| C4UKK5_YERRU | A0A085U923_YERRU | Chemotaxis protein CheY | -2.6 | **-9.3*** | **-9.6*** | **-3.5*** | **-3.6*** | -1.0 |
| C4UGW5_YERRU | A0A0A8V8I4_YERRU | Methyl-accepting chemotaxis (MCP) signaling domain protein | -2.0 | -2.8 | **-3.7*** | -1.4 | -1.8 | -1.3 |
| C4UG48_YERRU | A0A085U4X4_YERRU | PTS system, Lactose/Cellobiose specific IIB subunit | **-3.9*** | **-4.6*** | **-3.9*** | -1.2 | -1.0 | 1.2 |
| C4UFJ2_YERRU | A0A0A5FQB4_YERRU | Superoxide dismutase Cu-Zn | 2.8 | **3.2*** | **3.4*** | 1.2 | 1.2 | 1.1 |
| C4UH13_YERRU | A0A085U732_YERRU | Uncharacterized protein | 2.9 | 2.9 | **3.4*** | 1.0 | 1.2 | 1.2 |
| C4UM01_YERRU | A0A0A8VE52_YERRU | Glutaminase 1 | **6.7*** | **6.5*** | **8.2*** | -1.0 | 1.2 | 1.3 |
| A0A094SPR9_YERRU | A0A085U5L5_YERRU | Anti-anti-sigma factor family protein | **3.6*** | **3.7*** | **3.8*** | 1.0 | 1.1 | 1.0 |
| A0A094TJ61_YERRU | A0A085U489_YERRU | Allophanate hydrolase subunit 1 family protein | **-10.8*** | **-9.6*** | **-10.0*** | 1.1 | 1.1 | -1.0 |
| C4UIE6_YERRU | A0A085UB64_YERRU | Glycine cleavage system H protein | **-3.2*** | **-3.2*** | **-3.1*** | -1.0 | 1.0 | 1.0 |
| C4UJP9_YERRU | A0A085U5L7_YERRU | Anti-sigma regulatory factor (Ser/Thr protein kinase) | **3.9*** | **3.9*** | **4.3*** | -1.0 | 1.1 | 1.1 |
| C4ULB7_YERRU | A0A085U668_YERRU | PTS system, mannose-specific IIC component (EIIC-MAN) | -2.9 | -2.1 | **-4.8*** | 1.4 | -1.7 | -2.3 |
| C4UKH6_YERRU | A0A085U8Y4_YERRU | Flagellar motor switch protein FliM | **-3.4*** | **-3.9*** | **-6.0*** | -1.1 | -1.7 | -1.5 |
| C4UM00_YERRU | A0A085UBP7_YERRU | Amino acid permease-associated region | **4.3*** | **4.2*** | **4.0*** | -1.0 | -1.1 | -1.0 |
