# Supplementary material for: Shotgun proteomic analysis of Yersinia ruckeri strains under normal and iron-limited conditions
Source: Vet Res. 2016 Oct 6;47:100. doi: 10.1186/s13567-016-0384-3 (PMC5054536; doi:10.1186/s13567-016-0384-3)
Supplement: Supplementary file 6 — 10.1186/s13567-016-0384-3 Fold changes of differentially expressed proteins of Y. ruckeri strains compared to each other under iron-limited conditions. ANOVA was performed for UniProt database searches. * denotes statistically significant difference according to t test with FDR-adjusted p value <0.05 and fold change < −3 or > +3. [file 13567_2016_384_MOESM6_ESM.doc]

**Additional file 6 Fold changes of differentially expressed proteins of *Y. ruckeri* strains compared to each other under iron-limited culture conditions.** ANOVA was performed for UniProt database searches. * denotes statistically significant difference according to *t*-test with FDR-adjusted *p*-value < 0.05 and fold change < −3 or > +3.

| **UniProt  Accession number** | **Ambiguous Accession number** | **Protein** | **SP05**  **vs.**  **CSF007** | **SP05**  **vs.**  **7959** | **SP05**  **vs.**  **YRNC** | **CSF007 vs.**  **7959** | **CSF007 vs. YRNC** | **7959 vs. YRNC** |
| --- | --- | --- | --- | --- | --- | --- | --- | --- |
| C4UK36_YERRU | A0A085U984_YERRU | Ornithine decarboxylase, inducible | -2.9 | **-3.2*** | **-3.3*** | -1.1 | -1.1 | -1.0 |
| C4UML3_YERRU | A0A085UAK7_YERRU | Malic enzyme, NAD binding domain protein | **-3.3*** | **-3.8*** | **-3.5*** | -1.2 | -1.1 | 1.1 |
| A0A0A8VE48_YERRU | A0A085UBQ1_YERRU | Arginine deiminase | **6.0*** | **5.2*** | **6.6*** | -1.2 | 1.1 | 1.3 |
| C4UM15_YERRU | A0A0A8VGZ8_YERRU | Asparagine synthase | **-5.7*** | 1.0 | **-6.0*** | **5.8*** | -1.0 | **-6.0*** |
| C4UFF0_YERRU | A0A0A5FMC5_YERRU | Biodegradative arginine decarboxylase | **4.6*** | **6.2*** | **5.8*** | 1.3 | 1.2 | -1.1 |
| A0A085U669_YERRU | A0A085U669_YERRU | PTS mannose transporter subunit IIAB | -2.7 | -2.8 | **-3.2*** | -1.0 | -1.2 | -1.2 |
| C4UIW0_YERRU | A0A085U3M7_YERRU | Glycerol kinase | **-5.7*** | **-6.0*** | **-5.2*** | -1.1 | 1.1 | 1.2 |
| C4UMT2_YERRU | A0A085U9J0_YERRU | Aspartate ammonia-lyase | **-5.4*** | **-4.9*** | **-4.6*** | 1.1 | 1.2 | 1.1 |
| A0A0A8VDU4_YERRU | R4NIZ0_YERRU | Flagellar biosynthesis protein FliC | **-14.0*** | **-75.0*** | **-78.3*** | **-5.3*** | **-5.6*** | -1.0 |
| C4ULZ9_YERRU | A0A085UBP8_YERRU | Glutamate decarboxylase | **7.0*** | **7.4*** | **9.6*** | 1.1 | 1.4 | 1.3 |
| A0A0A5FJV0_YERRU | A0A085U4U1_YERRU | Catalase | **3.6*** | **3.8*** | **3.6*** | 1.1 | 1.0 | -1.0 |
| C4UNK2_YERRU | A0A085U9C1_YERRU | Malate dehydrogenase | -3.0 | -2.7 | **-3.0*** | 1.1 | -1.0 | -1.1 |
| C4UHI5_YERRU | A0A085U5C9_YERRU | Acriflavine resistance protein A | **3.3*** | **4.0*** | 2.4 | 1.2 | -1.4 | -1.7 |
| C4UH73_YERRU | A0A085U745_YERRU | Glucose-1-phosphate adenylyltransferase | **5.0*** | **5.6*** | **6.2*** | 1.1 | 1.2 | 1.1 |
| C4UNH4_YERRU | A0A0A8VK69_YERRU | Putative uncharacterized protein | **-4.4*** | **-6.5*** | **-7.2*** | -1.5 | -1.6 | -1.1 |
| C4ULZ7_YERRU | A0A085UBQ0_YERRU | Uncharacterized protein | **4.0*** | **3.0*** | **4.5*** | -1.3 | 1.1 | 1.5 |
| C4UNQ9_YERRU | A0A085UBM7_YERRU | Short chain dehydrogenase family protein | **6.1*** | **5.5*** | **5.7*** | -1.1 | -1.1 | 1.0 |
| C4UFT9_YERRU | A0A085U7X8_YERRU | Hemin transport protein hemS | **-3.7*** | **-3.9*** | **-3.6*** | -1.0 | 1.0 | 1.1 |
| C4UFT8_YERRU | A0A085U7X7_YERRU | Hemin receptor | **-4.3*** | **-4.3*** | **-5.1*** | 1.0 | -1.2 | -1.2 |
| C4UNS5_YERRU | A0A0A8VIU6_YERRU | Fumarate hydratase | **-3.1*** | **-3.2*** | **-3.1*** | -1.0 | 1.0 | 1.0 |
| C4UEU5_YERRU | A0A085U4B6_YERRU | DNA protection during starvation protein | 2.1 | 2.6 | **3.0*** | 1.3 | 1.5 | 1.2 |
| C4UG35_YERRU | A0A085U4W3_YERRU | HTH-type transcriptional regulator pecT | **-7.6*** | **-7.8*** | **-6.5*** | -1.0 | 1.2 | 1.2 |
| C4UKI6_YERRU | A0A0A8VHY4_YERRU | Flagellar hook protein FlgE | **-5.9*** | **-7.1*** | **-7.0*** | -1.2 | -1.2 | 1.0 |
| C4UFU0_YERRU | A0A085U7X9_YERRU | Hemin-binding periplasmic protein hmuT | **-3.3*** | **-3.8*** | **-3.5*** | -1.2 | -1.1 | 1.1 |
| A0A094V4E9_YERRU | A0A085U8W7_YERRU | Flagellin | **-24.1*** | **-102.9*** | **-91.1*** | **-4.3*** | **-3.8*** | 1.1 |
| A0A094V364_YERRU | A0A085UB63_YERRU | Aminomethyltransferase | **-3.8*** | **-3.6*** | **-3.5*** | 1.0 | 1.1 | 1.0 |
| C4UHA8_YERRU | A0A085U6U9_YERRU | 30S ribosomal protein S3 | 2.2 | **3.0*** | 1.8 | 1.4 | -1.2 | -1.7 |
| C4UJJ3_YERRU | A0A085U7G0_YERRU | Putative uncharacterized protein | **5.8*** | **5.4*** | **6.1*** | -1.1 | 1.1 | 1.1 |
| C4UMH7_YERRU | A0A085U484_YERRU | Pyroglutamyl-peptidase I | **-10.5*** | **-11.0*** | **-10.7*** | -1.0 | -1.0 | 1.0 |
| C4UG47_YERRU | A0A085U4X3_YERRU | Mannitol-specific cryptic phosphotransferase enzyme IIA component | -2.7 | **-3.8*** | **-3.1*** | -1.4 | -1.2 | 1.2 |
| C4UKK5_YERRU | A0A085U923_YERRU | Chemotaxis protein CheY | **-6.0*** | **-9.5*** | **-10.7*** | -1.6 | -1.8 | -1.1 |
| C4UHA5_YERRU | A0A085U6V2_YERRU | 50S ribosomal protein L2 | **3.5*** | **4.8*** | 2.1 | 1.4 | -1.7 | -2.3 |
| C4UG48_YERRU | A0A085U4X4_YERRU | PTS system, Lactose/Cellobiose specific IIB subunit | **-3.1*** | **-4.2*** | **-3.5*** | -1.3 | -1.1 | 1.2 |
| C4UGQ4_YERRU | A0A0A8VBV9_YERRU | ATP synthase subunit b | 1.6 | **3.4*** | 2.3 | 2.1 | 1.4 | -1.5 |
| C4UH13_YERRU | A0A085U732_YERRU | Uncharacterized protein | **3.2*** | 2.8 | **3.1*** | -1.1 | -1.0 | 1.1 |
| C4UNH3_YERRU | A0A085U928_YERRU | CheW-like domain protein | **-7.2*** | **-11.8*** | **-8.5*** | -1.6 | -1.2 | 1.4 |
| A0A094TL68_YERRU | A0A085U654_YERRU | Uncharacterized protein | **-3.5*** | **-4.3*** | **-3.5*** | -1.2 | 1.0 | 1.2 |
| C4ULL7_YERRU | A0A085U5U0_YERRU | Osmoprotectant transport protein OusBX | **3.4*** | 2.7 | **3.3*** | -1.2 | -1.0 | 1.2 |
| C4UIX0_YERRU | A0A085U3N7_YERRU | Periplasmic protein CpxP | **-5.2*** | **-3.2*** | **-4.6*** | 1.6 | 1.1 | -1.4 |
| A0A094T0U0_YERRU | A0A085U706_YERRU | Sporulation related domain protein | 2.9 | **5.3*** | 1.8 | 1.8 | -1.6 | -2.9 |
| C4UK00_YERRU | A0A0A8VFC3_YERRU | Putative uncharacterized protein | 2.8 | **5.2*** | 2.5 | 1.9 | -1.1 | -2.1 |
| C4UFW5_YERRU | A0A085U806_YERRU | 50S ribosomal protein L20 | 2.3 | **3.2*** | 1.9 | 1.4 | -1.3 | -1.7 |
| C4UJP9_YERRU | A0A085U5L7_YERRU | Anti-sigma regulatory factor (Ser/Thr protein kinase) | **3.2*** | 2.9 | **3.5*** | -1.1 | 1.1 | 1.2 |
| C4UP31_YERRU | A0A085U3I4_YERRU | Integral membrane protein | 2.4 | **3.9*** | 2.5 | 1.7 | 1.0 | -1.6 |
| C4UGI4_YERRU | A0A085UAB1_YERRU | Uncharacterized protein | **4.8*** | **4.8*** | **4.5*** | 1.0 | -1.1 | -1.1 |
| C4UM00_YERRU | A0A085UBP7_YERRU | Amino acid permease-associated region | **5.2*** | **8.5*** | **5.2*** | 1.6 | -1.0 | -1.7 |
